# Autophagy-deficient budding yeast cells are sensitive to freeze-thaw stress.

**DOI:** 10.17912/micropub.biology.000929

**Published:** 2025-03-27

**Authors:** Maria James, Grace K. Klain, Stacey O. Brito, Lupita Trejo, Teresa M. A. Okello, Verónica A. Segarra

**Affiliations:** 1 Department of Biological Sciences, Goucher College, Towson, Maryland, United States; 2 Department of Chemistry, Goucher College, Towson, Maryland, United States; 3 Department of Sociology, Goucher College, Towson, Maryland, United States

## Abstract

Autophagy enables eukaryotes to recycle damaged and unneeded materials to ensure survival in times of stress such as starvation. However, the full range of cellular stress responses that activate and require autophagy remains unknown. This study has compared the survival of wild type, *atg1Δ,* and *atg5Δ* budding yeast cells following freeze-thaw stress. The results indicate that cells deficient in autophagy exhibit enhanced sensitivity to freeze-thaw stress.

**Figure 1. atg1Δ and atg5Δ yeast cells exhibit increased sensitivity to freeze-thaw stress relative to wildtype:**
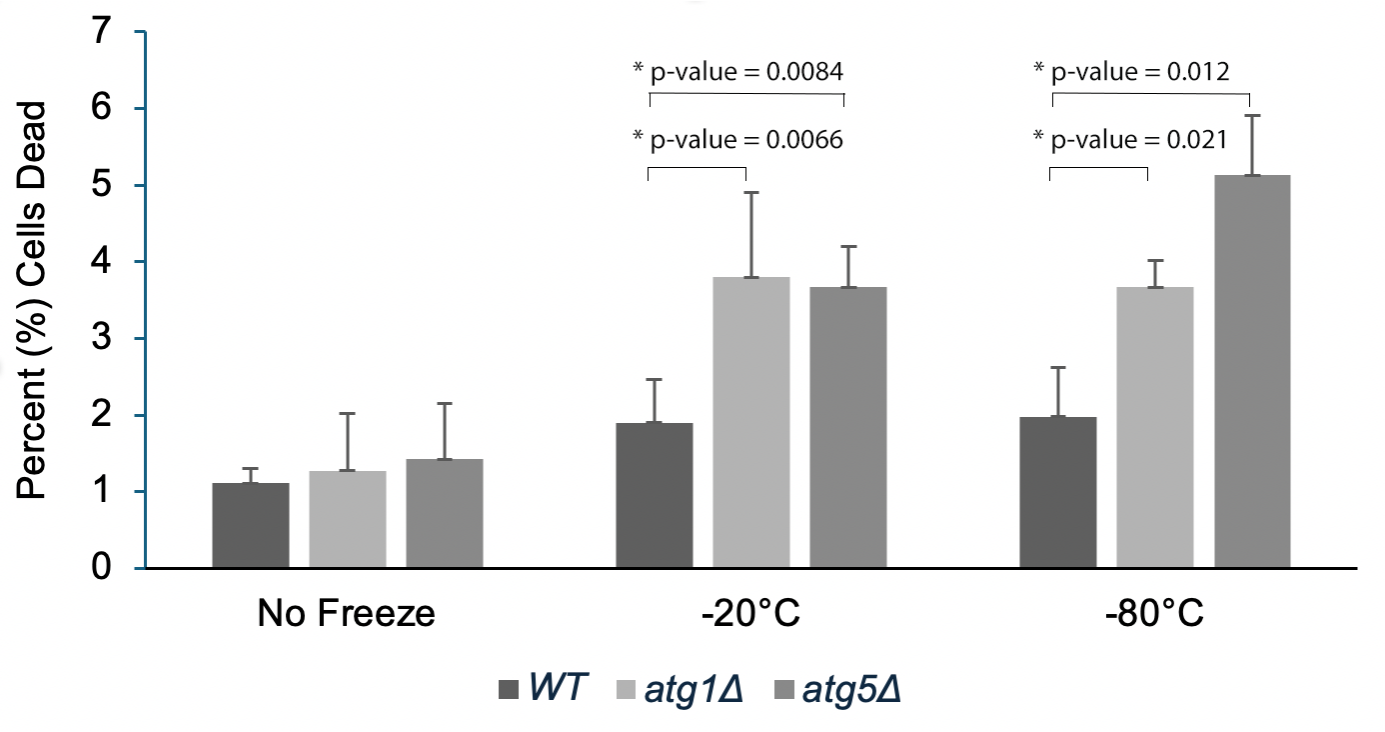
Cells were subjected to freeze-thaw stress and then trypan blue staining was used to calculate percent dead of wildtype (WT), *ATG1-* deleted ( *atg1Δ)* , and *ATG5-* deleted ( *atg5Δ)* yeast strains. Bar graph indicates average percent dead of four biological replicates. Error bars denote standard deviation and an asterisk indicates statistical significance assessed using two-tailed paired t-test. Brackets indicate the bars compared for statistical significance. Statistically significant p-values (less than 0.05) are indicated on the graph over the corresponding brackets.

## Description

Autophagy is an internal cellular recycling system that helps clear away damage within the cell and repurpose unneeded materials to ensure survival in times of stress. While a role has been identified for autophagy in the responses to nutrient, oxidative, Endoplasmic Reticulum, redox, and hypoxic stress among a few others (Kroemer et al., 2010), the full range of cellular stress responses that rely on autophagy remains unknown. This study has compared the survival of wildtype, *atg1Δ* , and *atg5Δ* budding yeast cells following freeze-thaw stress. *ATG1* codes for the Atg1p serine/threonine kinase responsible for autophagy induction, which leads to the formation of autophagosomes, the large vesicles unique to autophagy (Matsuura et al., 1997; Straub et al., 1997). *ATG5* codes for Atg5p, a component of a conjugation system that is unique to autophagy and plays a role in the expansion and completion of autophagosomes (Mizushima et al., 1998). Null mutations in *ATG1* have been shown to abrogate autophagy and are frequently used as a control for the absence of autophagy (Matsuura et al., 1997; Segarra et al., 2015). Null mutations in *ATG5* have also been shown to block autophagy in budding yeast (Kim et al., 2016).

The results indicate that *mutant cells* deficient in autophagy exhibit increased sensitivity to freeze-thaw stress. Figure 1 shows that both *atg1Δ* and *atg5Δ* yeast cells exhibit increased cell death relative to wildtype yeast upon freeze-thaw stress. This data suggests that autophagy is a contributing factor in yeast cells surviving freeze-thaw stress. Based on these findings, we hypothesize that autophagy is one of the cellular mechanisms used by cells to rid themselves of damage caused by freeze-thaw stress (Cabrera et al. 2020; Brito et al., 2024). Our findings are synergistic with observations by others where autophagic structures were associated with freezing stress in the aquatic *Lemna sp.* and nival (rocky habitats) *Ranunculus glacialis* plants, as well as *Acer saccharinum* (silver maple) samples *,* as determined by transmission electron microscopy (Steiner et al., 2020; Wesley-Smith et al. 2015). Additional studies are needed to ascertain the relationship between freeze-thaw stress and the triggering of autophagy.

## Methods

*Manipulation and growth of budding yeast.* Standard methods were used for manipulating and growing yeast (Guthrie and Fink 1991). The *S. cerevisiae* strains used in this study are listed in Table 1 below.

**Table 1.** *Yeast strains used in this study*

**Table:** 

**Alias**	**Genotype**	**Reference**
WT	*MATa his3Δ1 leu2Δ0 met15Δ0 ura3Δ0*	Giaever et al., 2002
*atg1Δ*	*MATα leu2 ura3-52 trp1 his3-Δ200 atg1Δ::natMX6*	Segarra et al., 2015
*atg5Δ*	*MATa his3Δ1 leu2Δ0 met15Δ0 ura3Δ0 atg5Δ::kanMX4*	Giaever et al., 2002

*Freeze-thaw stress sensitivity assays.* The protocol was adapted from Chen and Gibney (2022). In short, overnight yeast cultures in rich media (YEPD) were diluted to 3.0 OD _600_ units/mL, and 500 μL of yeast cell aliquots were transferred to microcentrifuge tubes. To assess viability before freezing, trypan blue staining was carried out (Strober 2015). Additional aliquots were pelleted by microcentrifugation, and cell pellets were frozen at either −20°C or −80°C. After 8 days at −20°C or −80°C, frozen cell pellets were resuspended in YEPD and stained with trypan blue to determine cell viability. The percentage of dead cells (% dead) was calculated and graphed (Figure 1).

*Statistical analyses.* Four individual biological replicates were carried out. The number of technical replicates within each biological replicate varied between one and three. The average of the biological replicates was calculated and plotted. Standard deviation was calculated and included in the graph as error bars. The p-value to assess statistical significance of difference between WT, *atg1Δ,* and *atg5Δ* was calculated using a two-tailed Student’s t-test.
